# Evaluation of 3D-Printed Polycaprolactone Scaffolds Coated with Freeze-Dried Platelet-Rich Plasma for Bone Regeneration

**DOI:** 10.3390/ma10070831

**Published:** 2017-07-19

**Authors:** Junda Li, Meilin Chen, Xiaoying Wei, Yishan Hao, Jinming Wang

**Affiliations:** Guangdong Provincial Key Laboratory of Stomatology, Guanghua School of Stomatology, Hospital of Stomatology, Sun Yat-sen University, Guangzhou 510055, China; lijda@mail2.sysu.edu.cn (J.L.); chenmlin@mail2.sysu.edu.cn (M.C.); weixying@mail2.sysu.edu.cn (X.W.); haoysh@mail2.sysu.edu.cn (Y.H.)

**Keywords:** 3D-printed scaffold, polycaprolactone, platelet-rich plasma, bone regeneration

## Abstract

Three-dimensional printing is one of the most promising techniques for the manufacturing of scaffolds for bone tissue engineering. However, a pure scaffold is limited by its biological properties. Platelet-rich plasma (PRP) has been shown to have the potential to improve the osteogenic effect. In this study, we improved the biological properties of scaffolds by coating 3D-printed polycaprolactone (PCL) scaffolds with freeze-dried and traditionally prepared PRP, and we evaluated these scaffolds through in vitro and in vivo experiments. In vitro, we evaluated the interaction between dental pulp stem cells (DPSCs) and the scaffolds by measuring cell proliferation, alkaline phosphatase (ALP) activity, and osteogenic differentiation. The results showed that freeze-dried PRP significantly enhanced ALP activity and the mRNA expression levels of osteogenic genes (ALP, RUNX2 (runt-related gene-2), OCN (osteocalcin), OPN (osteopontin)) of DPSCs (*p* < 0.05). In vivo, 5 mm calvarial defects were created, and the PRP-PCL scaffolds were implanted. The data showed that compared with traditional PRP-PCL scaffolds or bare PCL scaffolds, the freeze-dried PRP-PCL scaffolds induced significantly greater bone formation (*p* < 0.05). All these data suggest that coating 3D-printed PCL scaffolds with freeze-dried PRP can promote greater osteogenic differentiation of DPSCs and induce more bone formation, which may have great potential in future clinical applications.

## 1. Introduction

Bone injuries and defects caused by breaks, osteoporosis, pathological fractures, and tumors have resulted in thousands of surgeries. Autogenous bone grafts are still the gold standard of treatment. However, these grafts cannot be considered the best treatment for bone injuries and defects due to their obvious complications [1]. Bone tissue engineering—which involves a scaffold, seed cells, and growth factors—is now considered a more economical and effective approach to treating bone injuries and defects. The scaffold provides the structure and mechanical properties of the tissue-engineered bone. However, traditional methods of creating scaffolds (such as solvent casting, phase separation, fiber bonding, and gas formation) cannot fabricate polymeric scaffolds that meet the material requirements because these methods have limited control of the architecture and pore interconnectivity of the scaffold [2].

As 3D printing techniques continue to rapidly develop, the 3D-printed scaffold has shown a number of advantages and is gradually replacing the traditional scaffold used in cartilage and bone regeneration [3,4]. The 3D-printed scaffold is highly porous, with an interconnected pore structure that can transport nutrients and metabolic waste, and can be easily manipulated to form different shapes and sizes; thus, these scaffolds are suitable surfaces that are ideal for cell attachment and growth. The 3D-printed scaffold can also maintain suitable space for bone regeneration [5]. Polycaprolactone (PCL), an FDA-approved biocompatible material, is one of the most widely used polyester polymers in the field of bone tissue engineering [6]. Among its beneficial qualities, PCL can be readily fabricated and is biodegradable. Previous research has shown that PCL is also suitable for cartilage tissue engineering because it promotes cell attachment, proliferation, and matrix production [7,8,9]. Moreover, local PCL hydrolysis produces a minimal amount of acid compared with the hydrolysis of other polymers widely used in tissue engineering, such as polyglycolic acid (PGA) or polylactic acid (PLA) [10,11,12].

However, the bare PCL scaffold suffers from some drawbacks, such as the absence of biological properties, that limit cell-material interactions [13]. Therefore, many researchers have tried to modify the surface properties of PCL by improving its hydrophilicity or by introducing various functional groups to enhance its cell-material interaction [14,15].

Platelet-rich plasma (PRP) contains a cocktail of multiple growth factors, including platelet derived growth factor (PDGF), vascular endothelial growth factor (VEGF), transforming growth factor (TGF-β1), basic fibroblast growth factor (bFGF), epidermal growth factor (EGF), and insulin-like growth factor [16,17,18], and is frequently used in soft and bone tissue engineering [19,20,21,22]. Studies have shown that these growth factors strongly contribute to cell-extracellular matrix communication during regenerative activity [23]. In a previous study, PRP was used to facilitate the healing of damaged tissue [24]. PRP has also been applied in periodontal regenerative therapy of cartilage and osseous defects [25,26]. Another study proved that in vitro, PRP may potentially stimulate the proliferation and differentiation of mesenchymal stem cells (MSCs) in bone marrow [27]. To further expand the clinical applications of PRP, it was proven that biodegradable electrospun PCL scaffolds coated with PRP can improve the attachment and proliferation of MSCs compared to bare PCL fibers [28]. However, PRP is difficult to store due to its liquid form. To address this difficulty, some studies have used bovine thrombin and calcium chloride to turn liquid PRP into a solid form [29]. Recently, PRP was developed into a gel form that was termed platelet-rich fibrin (PRF). PRF is becoming increasingly popular, especially in the fields of clinical periodontology and oral maxillofacial surgery [30,31]. However, PRF failed to meet the requirements of clinical application in emergency care. Several studies have focused on developing a freeze-dried PRP, a solid form of PRP that not only solves the problem of storage but also maintains high levels of the growth factors it contains [32]. It has been proven that a biodegradable polymer material coated with freeze-dried PRP can improve the proliferation of human periodontal ligament cells in vitro and neovascularization in a chorioallantoic membrane assay [32].

Dental pulp stem cells, or DPSCs, are common candidates for scaffold-based tissue engineering [33]. DPSCs can be obtained more easily than bone marrow-derived stem cells (BMSCs). As neural crest-derived cells, DPSCs exhibit the characteristics of MSCs and have the ability to differentiate into other cells, as proved by our previous studies [34,35]. DPSCs have been proven to be able to differentiate into osteoblasts and to produce mineralized and extracellular matrix and bone-like trabecular structures [36,37]. Additionally, a recent study showed that DPSCs exhibit a higher osteogenic potency than BMSCs both in vitro and in a critical-size bone defect model [38]. DPSCs are thought to be a potentially useful source for bone regeneration due to their high rates of proliferation and differentiation [39].

We hypothesized that a 3D-printed PCL scaffold coated with freeze-dried PRP would improve the biological responses between the scaffold and the cells. In this study, we prepared the 3D-printed PCL scaffold by coating it with PRP via immersion, followed by freeze-drying. We examined the bone matrix formation around the scaffold in vitro using DPSCs seeded and cultured in the PRP-PCL scaffold; we assessed bone formation in vivo through the use of a critical-sized calvarial bone defect rat model to evaluate the effect of the PRP-PCL scaffold in bone regeneration.

## 2. Materials and Methods

### 2.1. Printing of 3D Scaffolds and Surface Treatment

The pure PCL scaffold was prepared with polycaprolactone powder (Cat# 25090, *M*_W_ = 50,000, Polysciences, Warrington, UK) and a 3D-Bioplotter (Envisiontec, 3D-Bioplotter, Gladbeck, Germany). The nozzle size and strand distance of the scaffold were 200 and 300 μm, respectively. The powder was placed in a steel syringe fastened to the printer and dispensed through a steel nozzle at temperatures >100 °C by applying air pressure (600 ± 25 kPa), and the feed rate was set at 80 mm/min. The PCL scaffolds were printed with a 5.0 mm diameter and were 1.0 mm in height. All scaffolds were sterilized with ethylene oxide before use [40]. Before coating with PRP, the scaffolds were first treated with ethanolic sodium hydroxide and 30% 0.25 M NaOH:70% absolute ethanol for 2 min to improve surface wettability [41]. Finally, the scaffolds were observed under a scanning electron microscope (SEM, Hitachi, S-450, Tokyo, Japan).

### 2.2. Preparation of Platelet-Rich Plasma

Platelet-rich plasma (PRP) was prepared from healthy, non-smoking volunteers aged 18–25 years using two methods: “Traditional PRP” and “Freeze-dried PRP”. In Traditional PRP, or the classical two-step centrifugation method [20], the blood was first centrifuged at 2400 rpm for 10 min so that we could separate PRP and platelet-poor plasma (PPP) portions from the red blood cell fraction. Next, the portions were centrifuged at 3600 rpm for another 15 min to concentrate the PRP. The upper 3/4 of the liquid was discarded, and the remainder was PRP. Eventually, we obtained approximately 1.5 mL of PRP from 8 mL of blood. In the freeze-dried PRP method, PRP was collected and activated using a freeze-thaw cycle [28]. First, PRP was stored at −80 °C for 24 h, stored at room temperature for 1 h, and then refrozen. After three cycles, PRP was centrifuged at 12,000 *g* for 10 min at 4 °C. The supernatant containing growth factors was recovered and kept frozen at −80 °C until use.

### 2.3. Coating Scaffolds with PRP

PCL scaffolds were immersed in PRP in a dish at room temperature for 5 min and then placed at −80 °C for 30 min. Next, the frozen samples were immediately freeze-dried using a Freeze-Dryer (CHRIST, Alpha 2-4 LD plus, Osterode, Germany). The freeze-dried PRP-PCL scaffold could be stored at 4 °C until use. Another group of PCL scaffolds (traditional PRP-PCL scaffolds) were immersed in PRP for 5 min at room temperature, and then 20 μL thrombin (100 IU/mL, Sigma, Darmstadt, Germany), and 20 μL 20% CaCl_2_ were mixed and loaded onto each PCL scaffold. These coated scaffolds were then placed in a 24-well plate.

### 2.4. Cell Culture

Human dental pulps were extracted from wisdom teeth of healthy, young adult volunteers undergoing orthodontic treatments according to a previously described protocol. After extraction, the teeth were cleaned and disinfected with 75% ethanol. The pulp chamber was exposed using sterilized dental fissure burs to obtain the pulp tissue, which was then stored in sterile tubes at 4 °C in Dulbecco’s Modified Eagle Medium (DMEM, Gibco, Carlsbad, CA, USA), 100 U/mL penicillin-G, and 100 μg/mL streptomycin. Each dental pulp was cut into pieces as small as possible under sterile conditions using a scalpel. The pulp pieces were transferred to a small capsule for digestion using collagenase type I. After 30 min, DMEM containing 5% FBS was added to stop the collagenase-mediated digestion. The suspension was collected and then filtered through a 70 μm cell strainer to obtain a homogenous suspension of DPSCs, which was then collected and centrifuged for 5 min at 1000 rpm. The cells were resuspended in DMEM, supplemented with 5% FBS, 100 U/mL penicillin-G, and 100 μg/mL streptomycin and were plated at 5 × 10^3^ cells/cm^2^. The cell culture medium was changed every two days. When the cells reached 80–90% confluence, we expanded the culture in the next passage. Passages III–V were used in the following tests.

### 2.5. In Vitro Experiments

#### 2.5.1. Scaffold Characterization

The morphology of the freeze-dried PRP-PCL scaffold, the traditional PRP-PCL scaffold and the bare PCL scaffold were observed under a scanning electron microscope. The samples were washed with PBS, fixed in glutaraldehyde, and dehydrated in an alcohol series. The scaffolds were dried and glued onto the holder, coated with gold under a vacuum using an ion coater, and then observed.

#### 2.5.2. Cell Attachment

The DPSCs were trypsinized and resuspended, and 1 × 10^5^ cells were seeded onto each scaffold. After 24 h, the culture medium was discarded and the scaffold was fixed in 4% paraformaldehyde solution for 30 min. Before staining with 100 nM rhodamine phalloidin (Cytoskeleton, Denver, CO, USA) for 30 min and 50 μmol/L 4’,6-diamidino-2-phenylindole (DAPI) for 10 min, the cells on the scaffold were treated with 0.1% Triton X-100 for 5 min and washed with PBS three times. Finally, the cell adhesion on the scaffold was observed using a fluorescence microscope (ZEISS, Axio observer Z1, Oberkochen, Germany).

#### 2.5.3. Cell Migration

DPSCs were cultured with DMEM without FBS for 24 h and were then seeded in a transwell chamber. The three groups of scaffolds were placed in a 24-well plate filled with DMEM supplemented with 5% FBS, 100 U/mL penicillin-G, and 100 μg/mL streptomycin. The transwell chamber was placed in the plate, and then 1 × 10^5^ DPSCs were resuspended with 200 μL DMEM and seeded into the transwell chamber. After 12 h, the chambers were washed with PBS and fixed in 95% alcohol for 15 min. The cells inside the chamber were discarded with a swab and the chambers were treated with 0.2% crystal violet for 15 min. After washing with PBS again, the number of cells at the bottom of the chamber was calculated from six samples using an optical.

#### 2.5.4. Cell Proliferation

The cellular viability of the samples was detected using the Cell Counting Kit-8 (CCK-8, Dojindo, Kumamoto, Japan) according to the protocol. Each scaffold was seeded with DPSCs adjusted to a population of 2000 cells/mL in 96-well plates. The cell-seeded scaffolds were incubated at 37 °C in 5% CO_2_ and the medium was replaced every two days. At 1, 3, 5, and 7 days, the culture medium was discarded and the cells were washed with PBS. A mixture consisting of 10 μL CCK-8 solution and 100 μL DMEM was added to the cells, which were then incubated for 2 h. The absorbance was measured at a 450 nm wavelength using a microplate reader (TECAN, Männedorf, Switzerland).

#### 2.5.5. Alkaline Phosphatase (ALP) Activity Assay

At 7, 14, and 21 days after the cells were seeded on the scaffolds, the samples were washed with PBS three times. 200 μL 1% Triton X-100 was added to lyse the cells on the scaffold overnight at 4 °C, and then the protein content of each sample was measured using a BCA Protein Assay Kit (CWBIO, Beijing, China). ALP activity was determined using the ALP Activity Assay Kit (Nanjing Jiancheng Bioengineering Institute, Nanjing, China) according to the protocol. Finally, the resultant absorbance was measured at a wavelength of 520 nm using a microplate reader.

#### 2.5.6. RNA Isolation and Analysis by Reverse Transcription Polymerase Chain Reaction (RT-PCR)

At seven and 14 days, the specimens were rinsed with PBS three times and total RNA was isolated using Trizol (Life Technologies, Waltham, MA, USA) and purified according to the manufacturer’s protocol. The RNA concentration and purity were determined using a NANODROP 2000c Spectrophotometer (Thermo Scientific, Waltham, MA, USA). The extracted RNA was reverse-transcribed into cDNA using a Prime ScriptTM RT Master Mix (Takara, Seoul, Korea). Quantitative real-time polymerase chain reaction (RT-PCR) was performed in triplicate using a LightCycler^®^ 480 (Roche Applied Science, Penzberg, Germany) and the LightCycler^®^ 480 SYBR Green I Master Kit (Roche Applied Science). The primers and probes for the reference gene GAPDH and for the target genes RUNX2, ALP, OPN and OCN were designed using Primer Premier 6 software (Table 1, Premier Biosoft, Palo Alto, CA, USA). The relative gene expression was quantified using the standard 2-(ΔΔC(t)) method.

### 2.6. In Vivo Bone Regeneration

#### 2.6.1. Surgery Procedures

**Figure 1:**
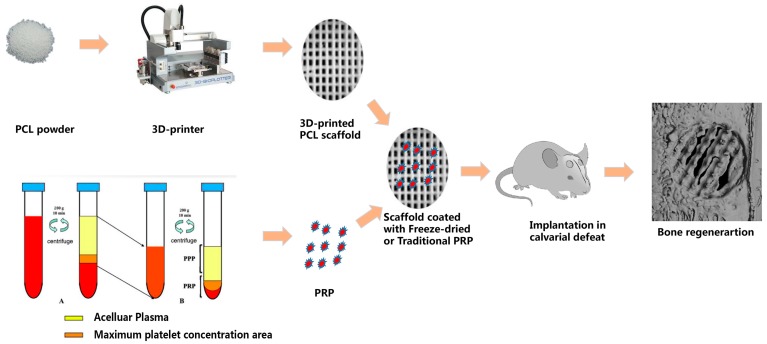
An overview of the in vivo bone regeneration of the calvarial defects.

The experimental protocol followed in this study was approved by the Animal Ethical and Welfare Committee of Sun Yat-Sen University. Bilateral 5 mm calvarial defects were created in 24 adult male Sprague-Dawley rats (200 ± 20 g). All animals were anesthetized via intraperitoneal injection of 10% chloral hydrate (300 mg/kg) and diethyl ether. The surgical site was sterilized using iodophor. The hair covering the calvarial bone was shaved and a full-thickness incision was made. The periosteum underlying the skull was sharply incised in order to sufficiently expose the bone for the trephine. The area was drilled using a trephine with cool saline to produce a 5 mm calvarial defect in both sides of the calvarial bone. The scaffolds were placed in the defects while another set of defects were left untreated to serve as controls. The incisions were closed with surgical sutures. The animals were kept in a warm place until they awoke and were set free to have food and water (Figure 1).

#### 2.6.2. Harvesting of Samples

The animals were sacrificed at 4, 8, and 12 weeks postoperatively. The calvarial bones were harvested and fixed in 4% paraformaldehyde solution for 48 h at 4 °C. The fixed samples were washed with PBS three times and were processed and analyzed histologically and by micro-computed tomography.

#### 2.6.3. Micro-CT Analysis

Each sample was assessed using a micro-computed tomography (Micro-CT) scanner (SCANCO μCT50, Muttenz, Switzerland) with a resolution of 20 μm, a source voltage of 70 kV and a current of 200 μA. Three-dimensional images were obtained and analyzed using Mimics software (Mimics 17.0, Materialise, Leuven, Belgium).

#### 2.6.4. Histological Staining

After the Micro-CT scan, the samples were decalcified with EDTA solution, dehydrated through a series of graded ethanol solutions, and embedded in paraffin. The samples were sliced into 5 μm sections. All sections were stained with hematoxylin-eosin staining and visualized using an optical microscope.

#### 2.6.5. Statistical Analysis

All experiments were carried out in triplicate. Analysis of variance (ANOVA) was applied to test the significance of the observed differences between the study groups. Differences were considered significant when *p* < 0.05.

## 3. Results

### 3.1. Characterization of 3D-Printed PCL Scaffolds after Surface Treatment

**Figure 2:**
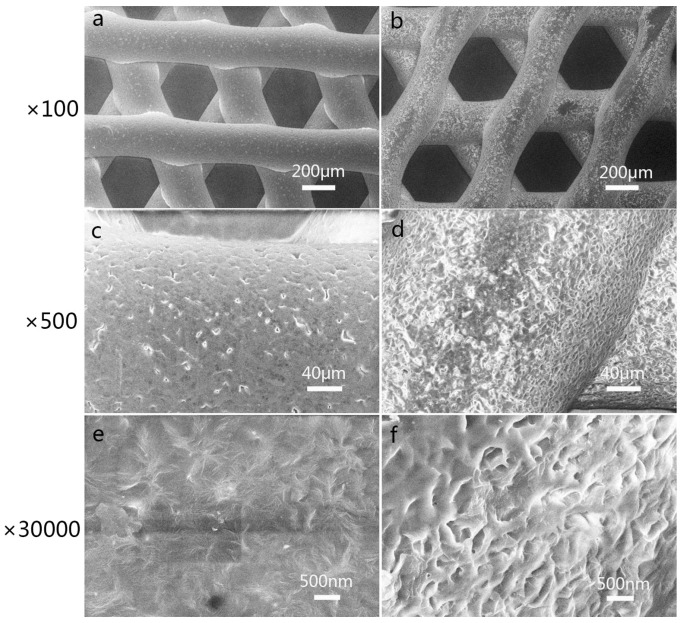
Characterization of 3D-printed polycaprolactone (PCL) scaffolds before (**a**,**c**,**e**) and after (**b**,**d**,**f**) treatment with ethanolic sodium hydroxide. At a higher magnification, we can see the roughness and micro-size pores (**d**,**f**). The magnification levels were ×100 (**a**,**b**), ×500 (**c**,**d**) and ×30,000 (**e**,**f**).

The surfaces of the scaffolds were observed by SEM, as shown in Figure 2. The bare scaffold in Figure 2a had a relatively smooth surface. After treatment with ethanolic sodium hydroxide, the surfaces of the scaffold became rough and had micro-size pores (Figure 2b,d,f), which were sufficient for the initial cell attachment.

### 3.2. Characterization of 3D-Printed PRP-PCL Scaffolds

**Figure 3:**
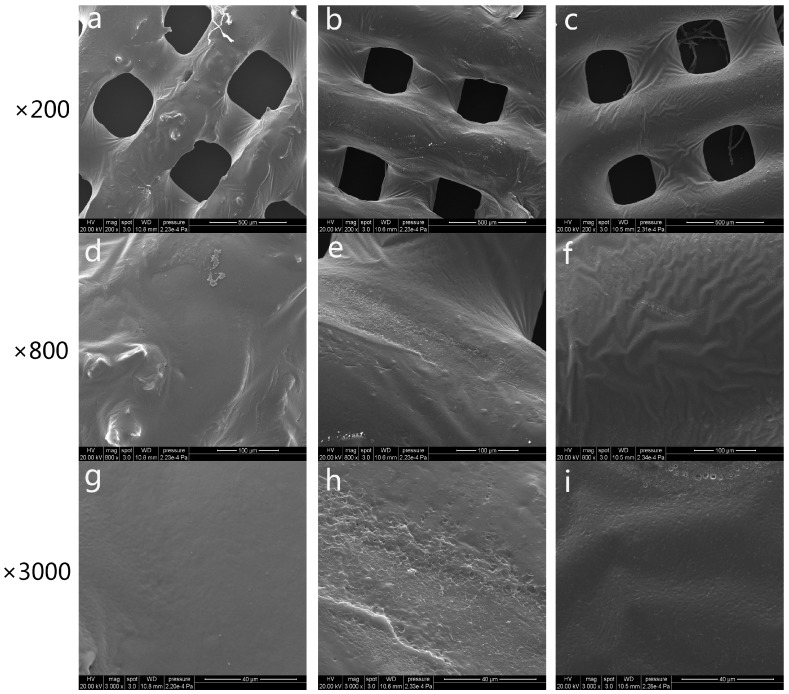
Scanning electron microscope (SEM) microphotographs of freeze-dried platelet-rich plasma polycaprolactone (PRP-PCL) scaffolds (**a**,**d**,**g**), traditional PRP-PCL scaffolds (**b**,**e**,**h**), and bare PCL scaffolds (**c**,**f**,**i**) at ×200, ×800, and ×3000 magnification. PRP could be seen after coating with freeze-dried PRP-PCL scaffolds (**a**,**d**,**g**) or traditional PRP-PCL scaffolds (**b**,**e**,**h**). Randomly distributed PRP are visible around the surface of the scaffolds, while no PRP are visible on the bare PCL scaffolds (**c**,**f**,**i**).

The morphology of the fabricated scaffolds was observed via SEM, as shown in Figure 3. The SEM observations revealed that the pore size of the scaffolds ranged from 250 to 300 μm. Compared with the traditional way of manufacturing the scaffold, 3D printing allows for much easier control of the pore size, interconnectivity, geometry of the pore structure, and reproduction of the resulting scaffold [42]. The bare PCL scaffolds shown in Figure 3c,f,i had relatively smooth surfaces. It has been proven that a smooth surface is not sufficient for cell attachment [43]. By coating the scaffold with PRP, we could see that the surface of the scaffold was absorbed by the PRP regardless of whether the PRP was freeze-dried (Figure 3a,d,g) or activated by CaCl2-thrombin (Figure 3b,e,h). Both coated scaffolds showed randomly distributed PRP around the scaffold surface. All scaffolds were observed at three different magnifications (×200, ×800, ×3000).

### 3.3. Effect of the PRP-PCL Scaffold on Cell Attachment

**Figure 4:**
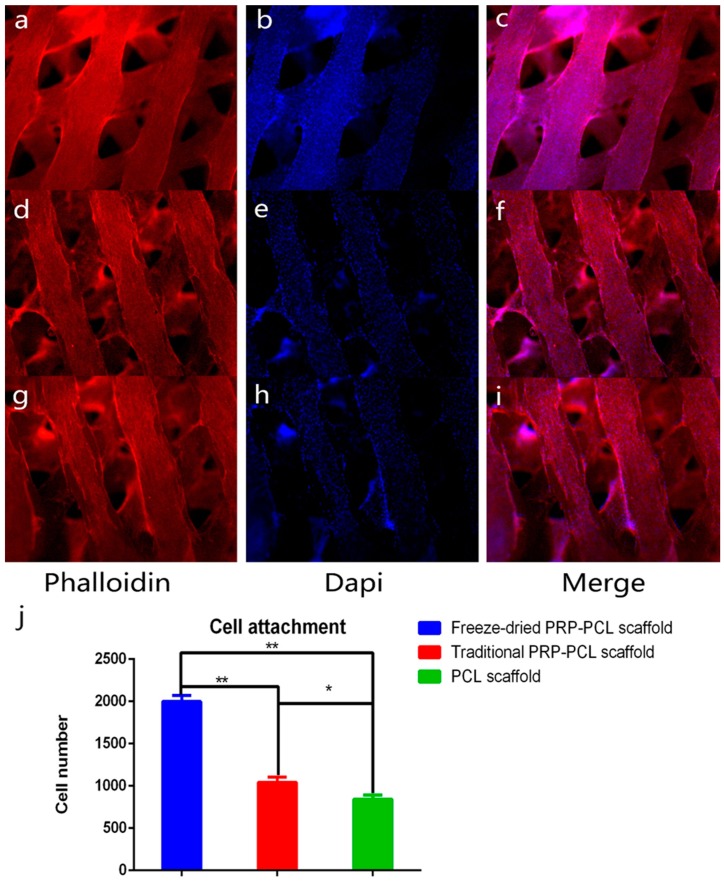
Cell attachment on the freeze-dried PRP-PCL scaffolds (**a**–**c**); traditional PRP-PCL scaffolds (**d**–**f**); bare PCL scaffolds (**g**–**i**) after three days of seeding; all images shown at ×50 magnification (* *p* < 0.05, ** *p* < 0.01) and (**j**) The number of the cell attachment.

DPSC nuclei were stained in blue, and the fibrin thread of the cells were stained in red. We calculated the number of cell nuclei using Image J software (Image J 2×, National Institutes of Health, Bethesda, MD, USA). As shown in Figure 4, we observed that the number of DPSCs on the PRP-PCL scaffolds was markedly higher than those on the bare PCL scaffolds. There was also a statistically significant difference between the freeze-dried PRP-PCL scaffold and the traditional PRP-PCL scaffold, which indicated that the freeze-dried PRP-PCL scaffold promoted more cell attachment. Coating the scaffold with PRP can promote scaffold wetting, which is beneficial for cell attachment. Moreover, the growth factors of PRP may endow the scaffold with high cell-loading properties.

### 3.4. Effect of the PRP-PCL Scaffold on Cell Migration

**Figure 5:**
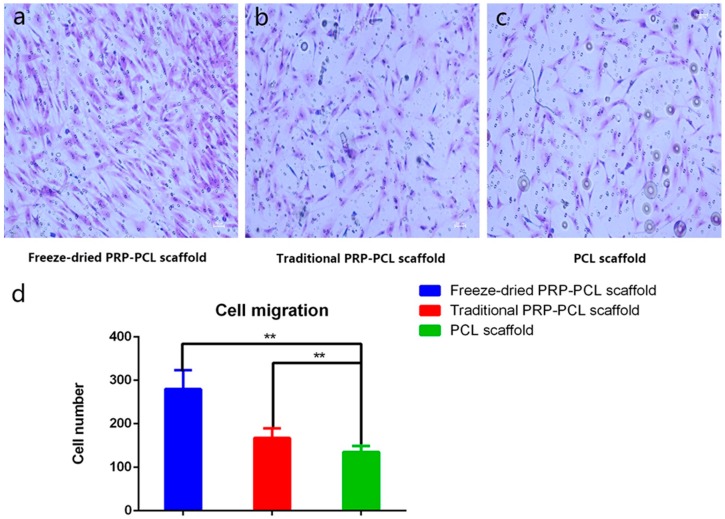
Number of migrated cells in the (**a**) freeze-dried PRP-PCL scaffold group; (**b**) traditional PRP-PCL scaffold group; (**c**) bare PCL scaffold group after 12 h of seeding; all images shown at ×50 magnification (** *p* < 0.01) and (**d**) The number of the cell migration.

The cells in six random samples were counted under an optical microscope at ×50 magnification. As shown in Figure 5, we visually observed that the number of migrated cells at the bottom of the transwell chamber in the freeze-dried PRP-PCL scaffold group was significantly higher than those of the traditional PRP-PCL scaffold group and the bare PRP-PCL scaffold group. There was no significant difference between the traditional PRP-PCL scaffold and bare PRP-PCL scaffold groups.

### 3.5. Effect of the PRP-PCL Scaffold on Cell Proliferation

**Figure 6:**
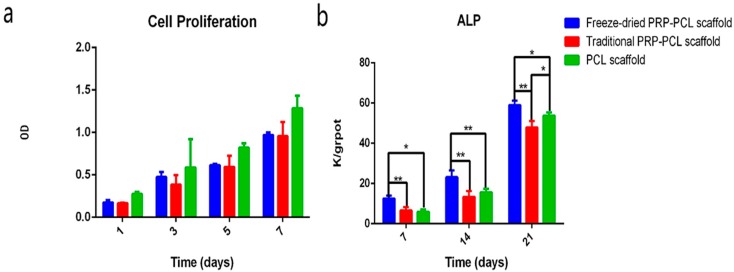
(**a**) Cell proliferation on the freeze-dried PRP-PCL scaffolds, traditional PRP-PCL scaffolds and bare PCL scaffolds 1, 3, 5, and 7 days after seeding; (**b**) ALP activity of the freeze-dried PRP-PCL scaffolds, traditional PRP-PCL scaffolds and bare PCL scaffolds seven and 14 days after seeding (* *p* < 0.05, ** *p* < 0.01).

In order to identify the effect of the PRP-PCL scaffolds on DPSC proliferation, we used the Cell Counting Kit-8 to test the DPSCs at 1, 3, 5, and 7 days after seeding on the scaffolds. As shown in Figure 6, we observed that as time progressed, cell proliferation could be seen in the PRP-PCL groups and in the bare PCL scaffold group. There was no statistically significant difference between each group on the same day (Figure 6a).

### 3.6. ALP Activity Assay

The ALP activity was evaluated using the ALP Activity Assay Kit at 7, 14, and 21 days. From the data shown in Figure 6b, we observed that the ALP activity of the cells cultured on scaffolds increased with an increasing number of days. The ALP activity of the freeze-dried PRP-PCL scaffold was significantly higher than those of the traditional PRP-PCL scaffold and the bare PCL scaffold (control) at 7, 14, and 21 days (*p* < 0.05). However, there were no statistically significant differences between the traditional PRP-PCL scaffold and the bare PCL scaffold except at 21 days (*p* < 0.05).

### 3.7. Differentiation of DPSCs: RT-PCR

**Figure 7:**
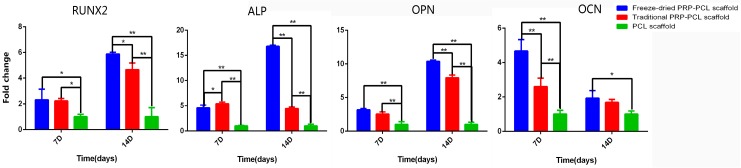
Expression of bone-specific genes, RUNX2, ALP, OPN, and OCN on the freeze-dried PRP-PCL scaffold, traditional PRP-PCL scaffold and bare PCL scaffold (* *p* < 0.05, ** *p* < 0.01).

The in vitro analyses of bone-specific gene expression are summarized in Figure 7. From the data, we observed that except for the expression of OCN by DPSCs on the traditional PRP-PCL scaffold at 14 days, the expression of RUNX2, ALP, OCN, and OPN by DPSCs on all PRP-PCL scaffolds at seven and 14 days were significantly higher than those on bare scaffolds. However, the level of OCN gene expression had contrasting results that showed that its expression was higher at 14 days than at seven days in all the scaffolds. The expression of ALP in the DPSCs on the traditional PRP-PCL scaffold was also higher at seven days than at 14 days. All of these differences were statistically significant except for the differences in expression of RUNX2 and OPN at seven days and OCN at 14 days between the freeze-dried PRP-PCL scaffolds and the traditional PRP-PCL scaffolds, which showed no statistical significance. The expression of OCN by DPSCs on both the traditional PRP-PCL scaffold and the bare PCL scaffold also showed the same result. At each time point, DPSCs cultured on the traditional PRP-PCL scaffold showed significantly higher expression of ALP, RUNX2, and OPN genes than those cultured on bare PCL scaffolds; moreover, data showed a better result that was statistically significant between the freeze-dried PRP-PCL scaffold and the bare PCL scaffold.

The gene expression levels of RUNX2, OCN, and OPN were highest in the freeze-dried PRP-PCL scaffolds, which indicated that the freeze-dried PRP promoted the osteogenic differentiation of DPSCs.

### 3.8. Micro-CT Analysis of the Critical-Size Calvarial Bone Defect in Rats

**Figure 8:**
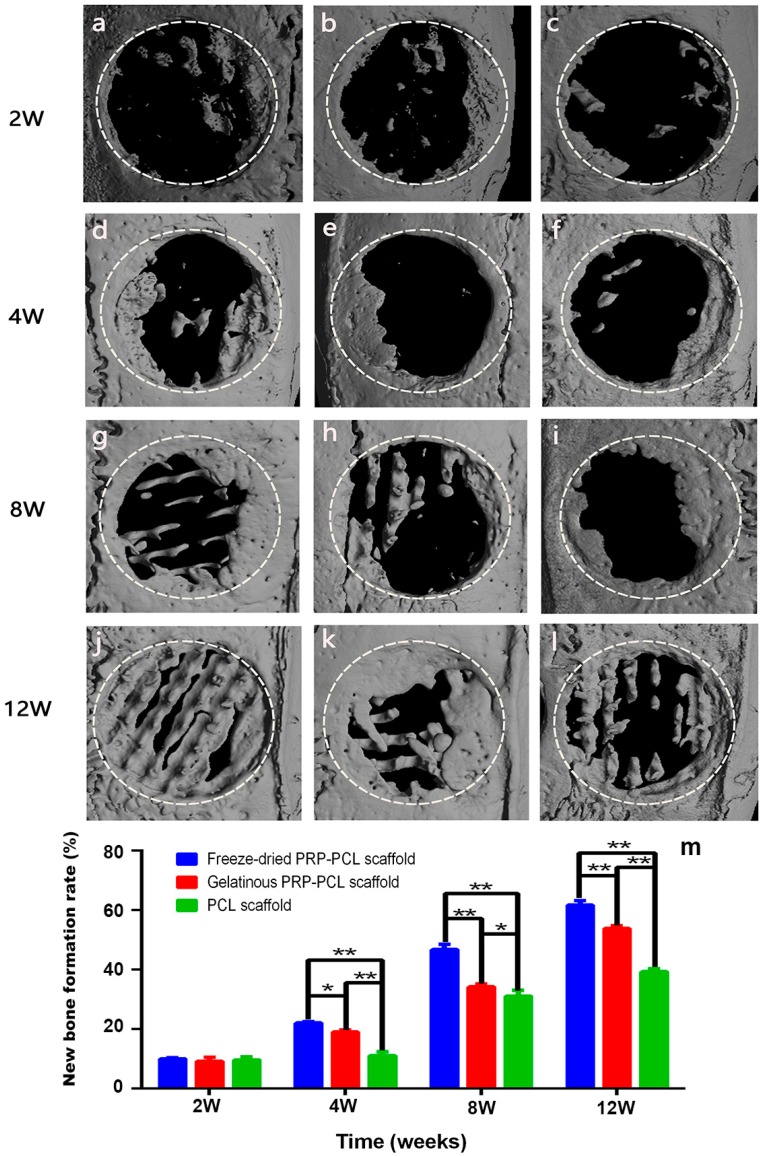
Evaluation of bone formation in calvarial defects via micro-CT. Representative micro-CT images of calvarial defects showing mineralized bone formation after treatment with freeze-dried PRP-PCL scaffolds (**a**,**d**,**g**,**j**), traditional PRP-PCL scaffolds (**b**,**e,h**,**k**) and bare PCL scaffolds (**c**,**f**,**i**,**l**). The bone tissue in the circle represents the regenerated bone. (**m**) Regenerated bone formation rate at 2, 4, 8 and 12 weeks after scaffold implantation (* *p* < 0.05, ** *p* < 0.01).

In order to evaluate the osteoinductive and osteoconductive potential of the 3D-printed PCL scaffolds coated with PRP, the freeze-dried PRP-PCL scaffolds, traditional PRP-PCL scaffolds and bare PCL scaffolds were implanted orthotopically on the critical-size calvarial bone defect in rats. All animals used in this experiment were healthy and were sacrificed 2, 4, 8, or 12 weeks after the implantation. Micro-CT analysis was performed using the samples obtained at these time points. The images showed that the defects of the bare PCL scaffold group remained largely open, with minimal mineralized new bone at the center of the defects or on regions confined mostly to the defect edges. Defects implanted with freeze-dried PRP-PCL scaffolds or the traditional PRP-PCL scaffolds showed more bone growth. Figure 8 shows that there was no significant difference among the three groups at two weeks. However, at 4, 8, and 12 weeks, the percentage of new bone formation in the experimental groups of both freeze-dried PRP-PCL scaffolds and the traditional PRP-PCL scaffolds were significantly higher than that of the bare PCL scaffold group. Compared with the traditional PRP-PCL scaffold group, the freeze-dried PRP-PCL scaffold group showed a significantly higher rate of new bone formation.

### 3.9. Histological Analysis Using H&E Staining

**Figure 9:**
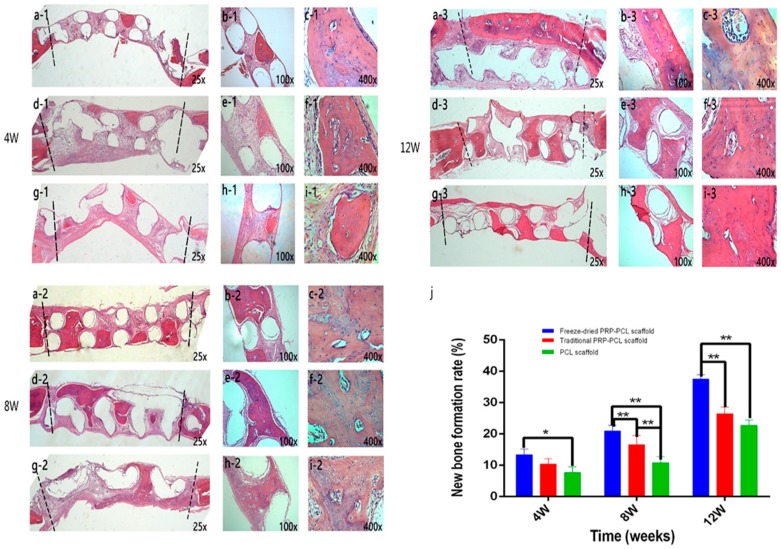
Histological sections stained with H&E showing calvarial defects treated with freeze-dried PRP-PCL scaffold (**a1**–**c1**,**a2**–**c2**,**a3**–**c3**), traditional PRP-PCL scaffold (**d1**–**f1**,**d2**–**f2**,**d3**–**f3**) and bare PCL scaffold (**g1**–**i1**,**g2**–**i2**,**g3**–**i3**) at 4, 8, and 12 weeks after implantation. The bone-like tissues between the dashed lines are newly formed bone. Empty circles are the scaffold locations. (**j**) New bone formation rate at 4, 8, and 12 weeks after scaffold implantation (* *p* < 0.05, ** *p* < 0.01).

To investigate new bone formation, histological sections of implants taken 4, 8, and 12 weeks after surgery were stained with H&E and then analyzed. In the bare PCL scaffold group at four weeks (Figure 9(g1–i1)), most regions of the defect were filled with connective fibrous tissue, and there was little new bone to be found. In the groups at eight and 12 weeks, more new bone could be seen in the defects. Compared with the bare PCL scaffold group, defects implanted with the freeze-dried PRP-PCL scaffold (Figure 9(a1–c1), (a2–c2) and (a3–c3)) or the traditional PRP-PCL scaffold (Figure 9(d1–f1), (d2–f2) and (d3–f3)) had more new bone formation at 4, 8, and 12 weeks post-implantation. The freeze-dried PRP-PCL scaffolds showed a faster rate of new bone formation than those of the traditional PRP-PCL scaffolds. At higher magnification, large amounts of bony nodules that can lead to the formation of thin strips of bony tissue could be seen (Figure 9(c1)). The morphological characteristics of normal bone were shown by the newly formed bone-like tissue. The figure shows that osteocytes were embedded in their lacuna and that osteoblasts lined the outer edge of the bone tissue. Moreover, the results of the H&E staining (Figure 9j), which were evaluated using the software Image-Pro Plus 6.0 (Media Cybernetics, Rockville, MD, USA), were similar to the micro-CT results.

## 4. Discussion

In this study, 3D-printed PCL scaffolds were used because the use of 3D printing technology allows for custom-designed scaffolds with controlled microstructures and interconnected porous structures [44,45]. With the rapid development of tissue engineering, 3D-printed scaffolds have seen widespread use in bone tissue engineering [46], cartilage tissue engineering [3], cardiac tissue engineering [47], and so on. In addition, PCL is one of the most commonly used thermoplastic polymers for 3D printing due to its prior FDA approval, biocompatibility, and biodegradability [48,49,50]. Moreover, its degradation by hydrolysis is relatively slow [51], so the use of a 3D-printed PCL scaffold for bone tissue engineering can be considered a good choice.

In order to evaluate the effect of the freeze-dried PRP on bone regeneration, we used both the freeze-thaw-freeze (FTF) cycle and CaCl2-thrombin to activate PRP and directly compared the osteogenesis effects of both types of PRP. The freeze-thaw-freeze (FTF) cycles were a relatively cheap and easy way to activate PRP while allowing it to retain the properties of proliferation and adhesion [52]. FTF promotes cytokine expression avoiding the use of CaCl_2_ or thrombin, which interfere with cell proliferation and differentiation. Moreover, it has been previously proven that after freeze-drying, the growth factors derived from frozen platelets still remain in viable condition [53].

The data indicated that the ALP activity of the freeze-dried PRP-PCL scaffold was significantly higher than that of the traditional PRP-PCL scaffold at 7, 14, and 21 days. Compared to the traditional PRP-PCL scaffold group, the freeze-dried PRP-PCL scaffold group showed significantly higher rates of new bone formation. All these data showed that the freeze-dried PRP method, which is the more convenient and efficient method of forming PRP, promoted greater bone formation than the traditional PRP method, which relies on activation of PRP via the use of CaCl_2_ or thrombin. A recent study showed that compared with the traditional PRP, freeze-dried PRP retained comparatively more growth factors after storage for four weeks at room temperature [54]. Another study showed that compared with traditional PRP, freeze-dried PRP released more growth factors, such as PDGF-BB and TGF-b1, and induced more bone formation in vivo [55]; these features could further improve the advantages of the freeze-dried PRP.

Our results clearly indicated that coating 3D-printed PCL scaffolds with freeze-dried PRP can promote mineralization and osteogenesis in vivo. This result may be attributed to the growth factors derived from PRP, which include vascular endothelial growth factor (VEGF), platelet derived growth factor (PDGF), basic fibroblast growth factor (bFGF), transforming growth factor (TGF-β1), epidermal growth factor (EGF), and insulin-like growth factor. However, the mechanism is not completely understood. The results found in this study may provide new treatment options that involve using 3D-printed PCL scaffolds in bone tissue engineering. However, the assessed explantation time points were 4, 8, and 12 weeks, which were considered relatively short; thus, an investigation over a longer time period, such as 16 weeks, may be required.

Further studies will be carried out to investigate the mechanisms by which PRP promotes mineralization and osteogenesis in vitro and in vivo. Additionally, it would be interesting to combine synthetic and natural materials instead of using a single material to manufacture scaffolds, thereby improving the bone regeneration capability.

## 5. Conclusions

We coated 3D-printed PCL scaffolds with freeze-dried PRP or traditional PRP and compared their relative abilities to drive osteoinduction in vitro and in vivo. Compared with the traditional PRP-PCL scaffolds and the bare PCL scaffolds, the freeze-dried PRP-PCL scaffolds exhibited greater ability to promote osteoinduction. The freeze-dried PRP also induced significant increases in ALP activity and the gene expression of ALP, OCN, RUNX2, and OPN compared with the traditional PRP-PCL and bare PCL scaffolds. Moreover, the developed freeze-dried PRP-PCL scaffolds showed better properties in terms of their ability to orthotopically increase bone regeneration. These results indicated that coating 3D-printed PCL scaffolds with freeze-dried PRP might better support bone regeneration than either traditional PRP-PCL scaffolds or bare PCL scaffolds.

## Figures and Tables

**Table 1:** PCR Primers for ALP, RUNX2, OCN, OPN, and GAPDH.

Gene	Prime Sequence (F, Forward; R, Reverse; 50–30)	Product Size (bp)
ALP	F: GCAACTTCCAGACCATTGGC R: TCCCACTGACTTCCCTGCTT	119
RUNX2	F: CGTGGCCTTCAAGGTGGTAG R: GAGGCATTCCGGAGCTCAG	105
OCN	F: AGCAAAGGTGCAGCCTTTGT R: GCGCCTGGGTCTCTTCACT	63
OPN	F: ACATCCAGTACCCTGATGCTACAG R: TGGCCTTGTATGCACCATTC	81
GAPDH	F: GATTCCACCCATGGCAAATT R: TCTCGCTCCTGGAAGATGGT	95
